# Pretreatment of the ROS Inhibitor Phenyl-N-tert-butylnitrone Alleviates Sleep Deprivation-Induced Hyperalgesia by Suppressing Microglia Activation and NLRP3 Inflammasome Activity in the Spinal Dorsal Cord

**DOI:** 10.1007/s11064-022-03751-5

**Published:** 2022-09-14

**Authors:** Yulin Huang, Jing Hao, Xuli Yang, Li Xu, Yue Liu, Yu’e Sun, Xiaoping Gu, Wei Zhang, Zhengliang Ma

**Affiliations:** 1 Nanjing Drum Tower Hospital Clinical College, Nanjing Medical University, Nanjing, China; 2 Department of Anesthesiology, Nanjing Drum Tower Hospital, The Affiliated Hospital of Nanjing University Medical School, Nanjing, China

**Keywords:** Sleep deprivation, Chronic postsurgical pain, Reactive oxygen species, NLRP3, Microglia activation

## Abstract

Sleep deprivation, a common perioperative period health problem, causes ocular discomfort and affects postsurgical pain. However, the mechanism of sleep deprivation-induced increased pain sensitivity is elusive. This study aims to explore the role of ROS in sleep deprivation (SD)-induced hyperalgesia and the underlying mechanism. A 48-h continuous SD was performed prior to the hind paw incision pain modeling in mice. We measured ROS levels, microglial activation, DNA damage and protein levels of iNOS, NLRP3, p-P65 and P65 in mouse spinal dorsal cord. The involvement of ROS in SD-induced prolongation of postsurgical pain was further confirmed by intrathecal injection of ROS inhibitor, phenyl-N-tert-butylnitrone (PBN). Pretreatment of 48-h SD in mice significantly prolonged postsurgical pain recovery, manifesting as lowered paw withdrawal mechanical threshold and paw withdrawal thermal latency. It caused ROS increase and upregulation of iNOS on both Day 1 and 7 in mouse spinal dorsal cord. In addition, upregulation of NLRP3 and p-P65, microglial activation and DNA damage were observed in mice pretreated with 48-h SD prior to the incision. Notably, intrathecal injection of PBN significantly reversed the harmful effects of SD on postsurgical pain recovery, hyperalgesia, microglial activation and DNA damage via the NF-κB signaling pathway. Collectively, ROS increase is responsible for SD-induced hyperalgesia through activating microglial, triggering DNA damage and enhancing NLRP3 inflammasome activity in the spinal dorsal cord.

## Introduction

There are up to 300 million surgical procedures annually in the world [1]. About 10–50% of surgical patients suffer continuous postsurgical pain [2], of which, about 2–10% is severe pain that lasts for at least one year [3]. Postoperative pain that persists for 3 months or longer is known as chronic postsurgical pain (CPSP), which is a common surgical outcome leading to long-term use of analgesics and lower health-related quality of life [4]. Risk factors of CPSP are diverse, including acute postsurgical pain, genetic susceptibility, preceding pain, age, sex, and psychosocial factors (e.g., perioperative sleep disorder, fear, and anxiety) [5]. Recent research has highlighted the negative influence of perioperative sleep deprivation (SD) on CPSP [6]. SD significantly causes hyperalgesia, and it further enhances pain facilitation [7]. Therefore, SD is considered as an etiological factor of chronic pain.

Short-term SD results in the increased levels of systemic reactive oxygen species (ROS), followed by serious physiological changes [8, 9]. During bedtime, sleep is responsible for fighting against oxidative stress, thus maintaining the normal function of the central nervous system (CNS). SD, however, breaks the balance of prooxidants and antioxidants in the CNS, that is, excessive ROS overwhelm the antioxidant system [10]. The imbalance of ROS production and antioxidants further causes malfunction or structure modification of major cellular DNAs [11, 12]. During the progression of neurodegenerative diseases, ROS generated by activated microglia are the murderer for neuroinflammation [13]. Jun et al. [14] reported that increased ROS upregulate NLRP3 inflammasome via the NF-κB signaling pathway. Activation of NLRP3 inflammasome contributes to pain development [15]. The exact mechanism underlying the role of ROS in perioperative SD-induced neuroinflammation, and the following chronic postsurgical pain, still remains unclear.

In the current study, a 48-h continuous SD was performed prior to the hind paw incision pain modeling in mice. We examined whether ROS-induced activation of microglia and NLRP3 inflammasome in spinal dorsal cord attributed to prolonged pain recovery time, and whether SD-induced mechanical allodynia and activation of NLRP3 inflammasome could be attenuated by ROS inhibitor phenyl-N-tert-butylnitrone (PBN). Finally, we explored the molecular mechanisms of perioperative SD in exacerbating postsurgical pain.

## Materials and Methods

### Animals

C57BL/6J mice with 8 weeks old (Beijing Vital River Laboratory Animal Technology, Beijing, China) were housed in the animal facility at a controlled temperature (21 ± 1 °C) with a standard 12-h light/dark cycle. They had free accesses to laboratory water and food pellets. In vivo experiments were approved by the Laboratory Animal of the Ethics Committee of Nanjing Drum Tower Hospital. They were randomly assigned into control group (con group), incisional pain model group (I group), SD procedure group (SD group) and SD procedure + incisional pain model group (SD + I group). In addition, mice intervened by SD procedures and incisional pain modeling were intrathecally injected with 100 µg/5 µl PBN (SD + I + PBN group) or isodose vehicle (SD + I + Veh group) during SD period and the day before making the incision.

### SD Procedures

The continuous 48-h SD was performed using a SD instrument (XR-XS108, Xinruan, Shanghai) with minor modifications of previously reported procedures [16]. Briefly, mice were placed in a cage, where a sweeper bar was moved along the bottom. An electrical motor system was used to control the speed, torque, and interval of the intermittent/continuous functioning mode of the sweeper, thus eliminating human errors. Mice needed to step over the sweeper and then resumed their unrestrained behaviors during the sweeper motion, and those maintained in the cage for continuous 48 h before the hind paw incision surgery were given food and water ad libitum.

### Incisional Pain Model in Mice

The plantar incisional surgery was performed as previously reported [17]. Briefly, mice were anesthetized with sevoflurane delivered via a small nose cone. The plantar aspect of the right hind paw was sterilized with a 10% povidone–iodine solution. A 0.5-cm longitudinal incision was made through the skin and fascia of the planter aspect of hind paw. The incision started from the edge of the heel and extended toward the toes. The plantar muscle was exposed, elevated and incised longitudinally. The skin was sutured with 5-0 nylon thread after hemostasis with gentle pressure. Mice were allowed to recover in their facilities postoperatively.

### Behavioral Analysis

Mechanical allodynia and paw withdrawal latency to a noxious heat stimuli were tested prior to SD (baseline), Day -2 and -1 during SD procedures, and Day 1, 3, 5, 7, 9 and 14 after incisional surgery.

### Paw Withdrawal Mechanical Threshold (PWMT)

Mechanical allodynia was performed by a series of von Frey filaments (Stoelting, USA). Briefly, mice were placed into individual transparent plexiglass compartments (length × width × height: 10 × 10 × 15 cm) onto a metal mesh floor (graticule: 0.5 × 0.5 cm). After acclimatized for at least 30 min, Von Frey filaments (0.16, 0.4, 0.6, 1.0, 1.4, and 2.0 g) were vertically poked into the plantar surface of the right hind paw and held in place for 6–8 s, which was tested for five times. The lowest stimulus strength that caused brisk withdrawal of the paw or paw flinching that produced three or more positive responses in total five times was regarded as PWMT.

### Paw Withdrawal Thermal Latency (PWTL)

PWTL to a noxious heat stimulus was measured by the Model 336 Analgesic Meter (IITC Inc./Life Science Instruments, Woodland Hills, USA). Briefly, mice were placed in an individual plexiglass compartments (length × width × height: 10 × 10 × 15 cm) on a glass plate. The middle of the plantar surface of the right hind paw receipted a radiant heat produced by a light beam through the glass plate, which was turned off after foot lifting. The duration from the start of the light beam and foot lifting was recorded as PWTL. Each test was repeated five times with a 5-min interval. To avoid tissue damage to the hind paw, a cut-off time of 25 s was necessary.

### Drug Administration

N-tert-Butyl-α-phenylnitrone (PBN, Sigma-Aldrich, St. Louis, MO, USA) was dissolved in 0.9% saline (20 μg/μl). Mice were treated with 100 μg/5 μl PBN or saline by intrathecal injection (i.t.) into the mouse lumbar subarachnoid space at the L5–L6 intervertebrae for continuous 3 days, according to the modification of a method described previously [18]. The plantar incisional surgery was performed half an hour after the third administration of PBN.

### Western Blotting

Mice were sacrificed under overdose sevoflurane. Tissue proteins from the entire lumbosacral enlargement of spinal cord was extracted and homogenized in ice-cold RIPA Lysis Buffer containing protease inhibitor cocktail and lysates. Tissue samples were then centrifuged at 4 °C, 12,000 rpm for 15 min, and the supernatants were collected for further examination. The protein concentration was determined using an enhanced BCA Protein Assay Kit (P0010, Beyotime, China). A total of 20 μg protein of each sample was separated by an appropriate concentration of SDS-PAGE (PG-112, Epizyme Biotech, China) and then transferred onto polyvinylidene fluoride (PVDF) membranes (IPVH00010, Millipore, USA). The membranes were first blocked with 5% non-fat milk for 1 h at room temperature (RT) and then incubated with indicated primary antibodies against NLRP3 (ab263899, 1:1000, RRID:AB_9890, Abcam, USA), iNOS (ab178945, 1:1000, RRID:AB_2861417, Abcam, USA), P65 (#8242, 1:1000, RRID:AB_10859369, CST, USA), p-P65 (#3033, 1:1000, RRID:AB_331284, CST, USA), and β-actin (bs-0061R, 1:5000, RRID:AB_10855480, Bioss, China) overnight at 4 °C. The membranes were incubated with goat anti-rabbit IgG secondary antibody (A0208, 1:1000, RRID:AB_2892644, Beyotime, China) for 1 h at RT after washing with TBST. Finally, Protein bands were visualized with ECL Prime Detection Reagent (E412-02, Vazyme, China). Images were captured using a cooled CCD system (4600SF, Tanon, China). Quantitative densitometry analysis was performed using Image J (NIH, Bethesda, MD).

### Immunofluorescence Staining

Under deep anesthesia with sevoflurane, the entire lumbosacral enlargement was quickly removed, fixed with 4% paraformaldehyde, and dehydrated in 30% sucrose. The L3-L5 spinal cords were transversely cut into 25-μm-thick sections and mounted on glass slides. Sections were blocked and then incubated with primary antibodies against Iba-1 (#019-19741, 1:700, RRID:AB_839504, Wako, Japan) and 8-OHdG (ab62623, 1:500, RRID:AB_940049, Abcam, USA) overnight at 4 °C. After washing with phosphate-buffered saline (PBS), sections were incubated with Alexa 488-conjugated goat anti-rabbit antibody (A32731, 1:1000, RRID:AB_2633280, Thermo Fisher, USA) and Alexa 594-conjugated goat anti-mouse antibody (A32742, 1:1000, RRID:AB_2762825, Thermo Fisher, USA) in the dark. 3 of spinal cord sections per animal were employed for further statistical analysis. The fluorescence intensity of Iba1 and 8-OHdG were measured by Image J (NIH, Bethesda, MD).

### Measurement of ROS Levels

The lumbosacral enlargement of spinal cord samples (200 mg) was washed with PBS, and homogenized in ice-cooled Tris–HCL buffer (40 mM, pH 7.4). A total of 100 μl of tissue homogenates were incubated with the diluted 10 μM DCFH-DA (S0033S, Beyotime, China) in Tris–HCL buffer (40 mM, pH 7.4) at 37 °C for 30 min. The fluorescence intensity was assessed by a multifunctional microplate reader (λexcitation = 488 nm and λemission = 525 nm) (Spark, Tecan, Switzerland).

### Statistical Analysis

All data were expressed as mean ± SD and evaluated by SPSS software 22.0. Changes of behavioral assessments were analyzed using two-way repeated measures of variance. Differences between two or more groups were compared by the unpaired two-tailed Student’s *t* test or one-way ANOVA, respectively, followed by the post hoc test (Bonferroni test). *P* value < 0.05 was accepted as significant.

## Results

### Pretreatment of Short-Term SD in Mice Prolongs Postsurgical Pain Recovery

**Fig. 1:**
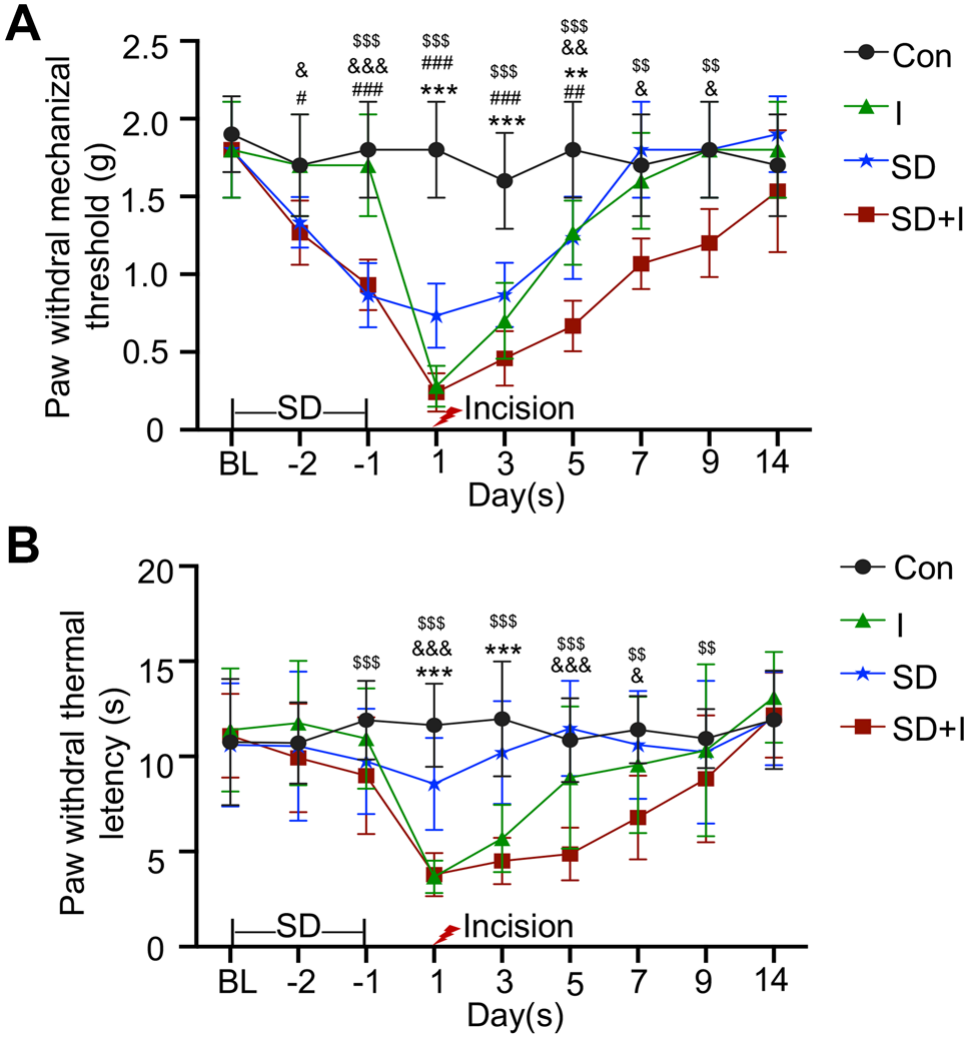
Time-dependent changes in paw withdrawal mechanical threshold and thermal latency after continuous 48 h sleep deprivation in mice. **A** Paw withdrawal thresholds in response to mechanical stimulation during SD (Day -2 and -1) and on the days post-incision (Day 1, 3, 5, 7, 9, and 14). **B** Paw withdrawal to heat stimulation in corresponding time points. Two-way repeated ANOVA followed by the Bonferroni post hoc test. n = 8 per group. Con vs. I: **p < 0.01, ***p < 0.001; Con vs. SD: ^#^p < 0.05, ^##^p < 0.01,^###^p < 0.001; Con vs. SD + I: ^$$^p < 0.01,^$$$^p < 0.001; I vs. SD + I: ^&^p < 0.05, ^&&^p < 0.01,^&&&^p < 0.001

In the present study, a continuous 48-h SD was performed prior to the unilateral hind paw plantar incision, aiming to explore the influence of pretreatment of short-term SD on postsurgical pain recovery. Baseline measurements were comparable in con group, I group, SD group and SD + I group. Compared with mice in con group, the incision resulted in persistent mechanical, thermal, and heat pain hypersensitivities on the ipsilateral side of mice in I group. Pain hypersensitivity in I group peaked on Day 1, then gradually recovered, and completely disappeared on Day 7 [con group vs*.* I group: PWMT: F(3,21) = 38.681, *p* < 0.001 for group factor, F(8,56) = 48.139, *p* < 0.001 for time factor, F(24,168) = 7.795, *p* < 0.001 for interaction; *p* < 0.001 on Day 1 and 3, *p* < 0.01 on Day 5; Day 1: F(3,21) = 73.929, *p* < 0.001 for group factor; Day 3: F(3,21) = 25.216, *p* < 0.001 for group factor; Day 5: F(3,21) = 21.798, *p* < 0.001 for group factor; PWTL: F(3,21) = 34.656, *p* < 0.001 for group factor, F(8,56) = 26.631, *p* < 0.001 for time factor, F(24,168) = 7.87, *p* < 0.001 for interaction; *p* < 0.001 on Day 1 and 3; Day 1: F(3,21) = 85.812, *p* < 0.001 for group factor; Day 3: F(3,21) = 43.43, *p* < 0.001 for group factor]. As expected, the continuous 48-h SD exposure significantly reduced PWMT during SD period, and lasted for 5 days following SD exposure [con group vs*.* SD group: F(3,21) = 34.656, *p* < 0.001 for group factor, F(8,56) = 35.742, *p* < 0.001 for time factor, F(24,168) = 4.570, *p* < 0.001 for interaction; *p* < 0.05 on Day -2, *p* < 0.001 on Day -1, 1, and 3, *p* < 0.01 on Day 5; Day -2: F(3,21) = 1.087, *p* = 0.361 for group factor; Day -1: F(3,21) = 4.211, *p* < 0.01 for group factor; Day 1: F(3,21) = 85.812, *p* < 0.001 for group factor; Day 3: F(3,21) = 43.43, *p* < 0.001 for group factor; Day 5: F(3,21) = 23.583, *p* < 0.001 for group factor). Compared with group I, the continuous 48-h SD aggravated and prolonged the postoperative pain of mice in group SD + I (I group vs*.* SD + I group: PWMT: *p* < 0.05 on Day -2, *p* < 0.001 on Day -1, *p* < 0.01 on Day 5, *p* < 0.05 on Day 7 and Day 9; Day -2: F(3,21) = 4.548, *p* = 0.014 for group factor; Day -1: F(3,21) = 21.358, *p* < 0.001 for group factor; Day 5: F(3,21) = 21.798, *p* < 0.001 for group factor; Day 7: F(3,21) = 7.857, *p* < 0.01 for group factor; Day 9: F(3,21) = 6.429, *p* < 0.05 for group factor) (Fig. 1A, B). Collectively, the above data suggested that pretreatment of short-term SD prolonged postsurgical pain recovery in mice with a unilateral hind paw plantar incision.

### Pretreatment of SD Enhances ROS Levels in the Spinal Cord

**Fig. 2:**
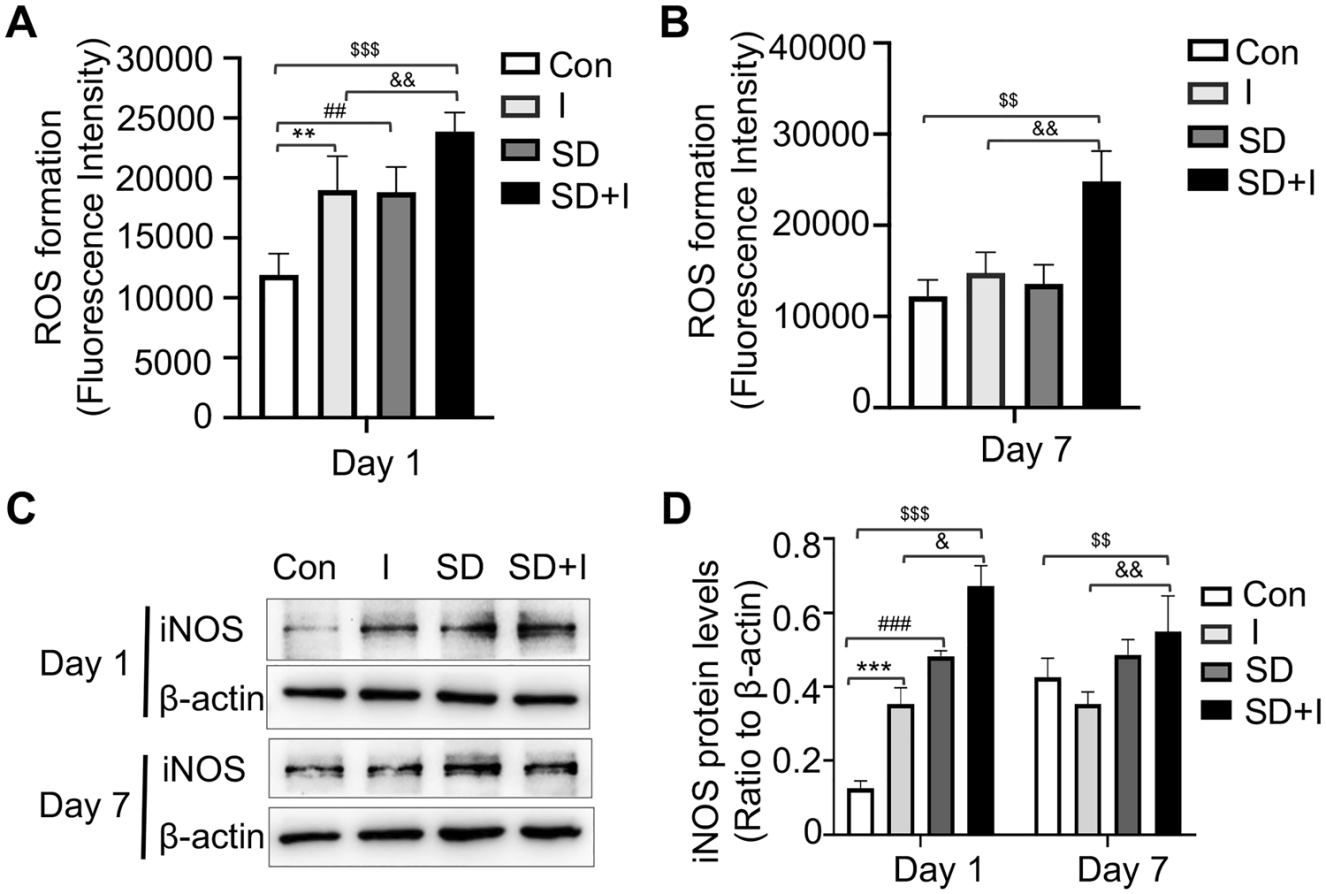
ROS levels and iNOS expression on Day 1 and Day 7. **A**, **B** Fluorescence intensity of ROS levels in spinal cord tissue of Con, I, SD, and SD + I group mice on day 1 and 7 post-incision. n = 4 per group. **C** iNOS expression in spinal cord on day 1 and 7 post-incision measured by western blot. n = 4 per group. **D** Quantification of iNOS protein levels. Two-way ANOVA for comparisons including more than two groups. Con vs. I: **p < 0.01,***p < 0.001; Con vs. SD: ^##^p < 0.01,^###^p < 0.001; Con vs. SD + I: ^$$^p < 0.01,^$$$^p < 0.001; I vs. SD + I: ^&^p < 0.05, ^&&^p < 0.01

ROS levels in mouse spinal cord were measured on Day 1 and 7. Compared with those of con group, ROS levels on Day 1 in the remaining groups all significantly increased [con group vs*.* I group, *p* < 0.01; con group vs*.* SD group, *p* < 0.01; con group vs*.* SD + I group, *p* < 0.001; I group vs*.* SD + I group, *p* < 0.01; F(3,12) = 21.228, *p* < 0.001 for group factor] (Fig. 2A). Consistently, ROS levels in SD + I group on Day 7 were still significantly higher than those of con group and I group [con group vs*.* group SD + I, *p* < 0.01; I group vs*.* SD + I group, *p* < 0.01; F(3,12) = 20.568, *p* < 0.001 for group factor] (Fig. 2B). It is suggested that pretreatment of SD increased ROS levels in mouse spinal cord, which, importantly, persistently remained high after the unilateral hind paw plantar incision. Here, we detected the protein level of iNOS in the spinal cord by Western blotting. It was significantly upregulated in I, SD and SD + I group on Day 1 (con group vs*.* I group, *p* < 0.001; con group vs*.* SD group, *p* < 0.001; con group vs*.* SD + I group, *p* < 0.001; I group vs*.* SD + I group, *p* < 0.05; F(3,12) = 51.139, *p* < 0.001 for group factor). Notably, the high protein level of iNOS lasted on Day 7 in SD + I group (con group vs*.* SD + I group, *p* < 0.01; I group vs*.* SD + I group, *p* < 0.01; F(3,12) = 11.298, *p* = 0.001 for group factor), which was consistent with the changes of ROS levels (Fig. 2C, D).

### SD-Induced Accumulation of ROS Triggers the Activation of Microglia and DNA Damage in Mouse Spinal Dorsal Cord

**Fig. 3:**
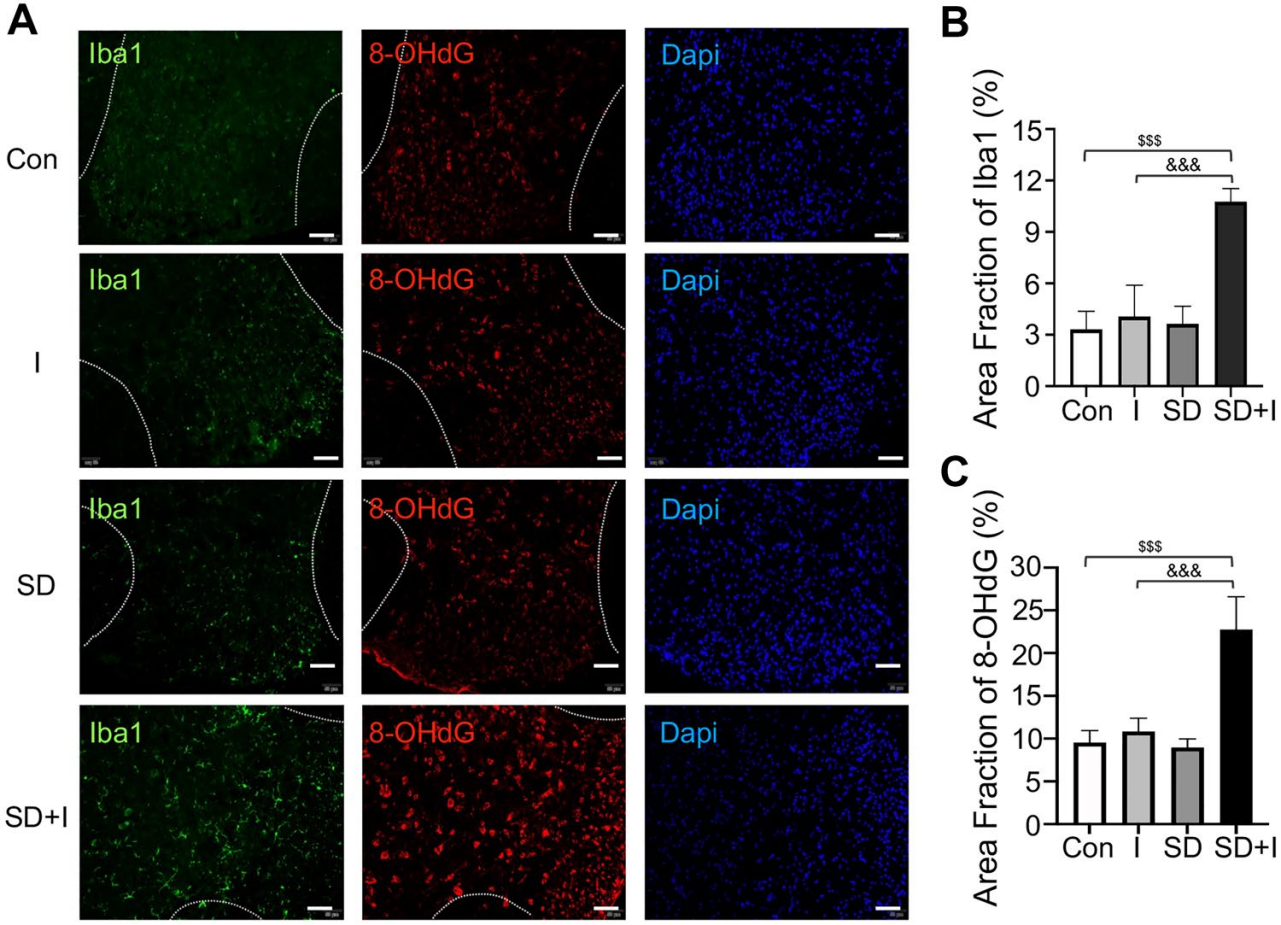
Effects of preoperative sleep deprivation on the activation of spinal microglia and DNA damage after incisional surgery. **A** Images of immunostaining for Iba1(microglia marker) and 8-OHdG (DNA damage marker) in lumbar spinal dorsal horn ipsilateral to incision were captured in group Con and group SD + I on Day 7 post-incision. Scale bar = 50 μm. n = 3 per group. **B**–**C**. Quantification of Iba1 and 8-OHdG fluorescence intensity, respectively. Two-way ANOVA for comparisons between four groups. Con vs. SD + I: ^$$$^p < 0.001, I vs. SD + I: ^&&&^p < 0.001

ROS are capable of triggering inflammatory response in the CNS. Activated microglia would produce harmful oxidizing agents, which further activate microglia in a vicious circle. On Day 7, positive expression of Iba1 in mouse spinal dorsal cord of SD + I group was significantly higher than that of con group (con group vs*.* SD + I group, *p* < 0.001; I group vs*.* SD + I group, *p* < 0.001; F(2,11) = 25.104, *p* < 0.001 for group factor;), suggesting the increased activation of microglia in SD + I group on Day 7(Fig. 3A). DNA single-strand break may result from various sources, including environmental factors like ROS. As shown in Fig. 3A, the expression of 8-OHdG (DNA single-strand break marker) in mouse spinal dorsal cord of SD + I group was significantly higher than that of con group on Day 7 (con group vs*.* SD + I group, *p* < 0.001; I group vs*.* SD + I group, *p* < 0.001; F(2,11) = 25.576, *p* < 0.001 for group factor;) While compared with Con group, the expression of Iba1 and 8-OHdG have no significance in I group and SD group, which was consistent with the changes in pain behavior. (Fig. 3A, B).

### SD-Induced Activation NF-κB/NLRP3 in Mouse Spinal Dorsal Cord Contributes to Prolong the Postsurgical Pain

**Fig. 4:**
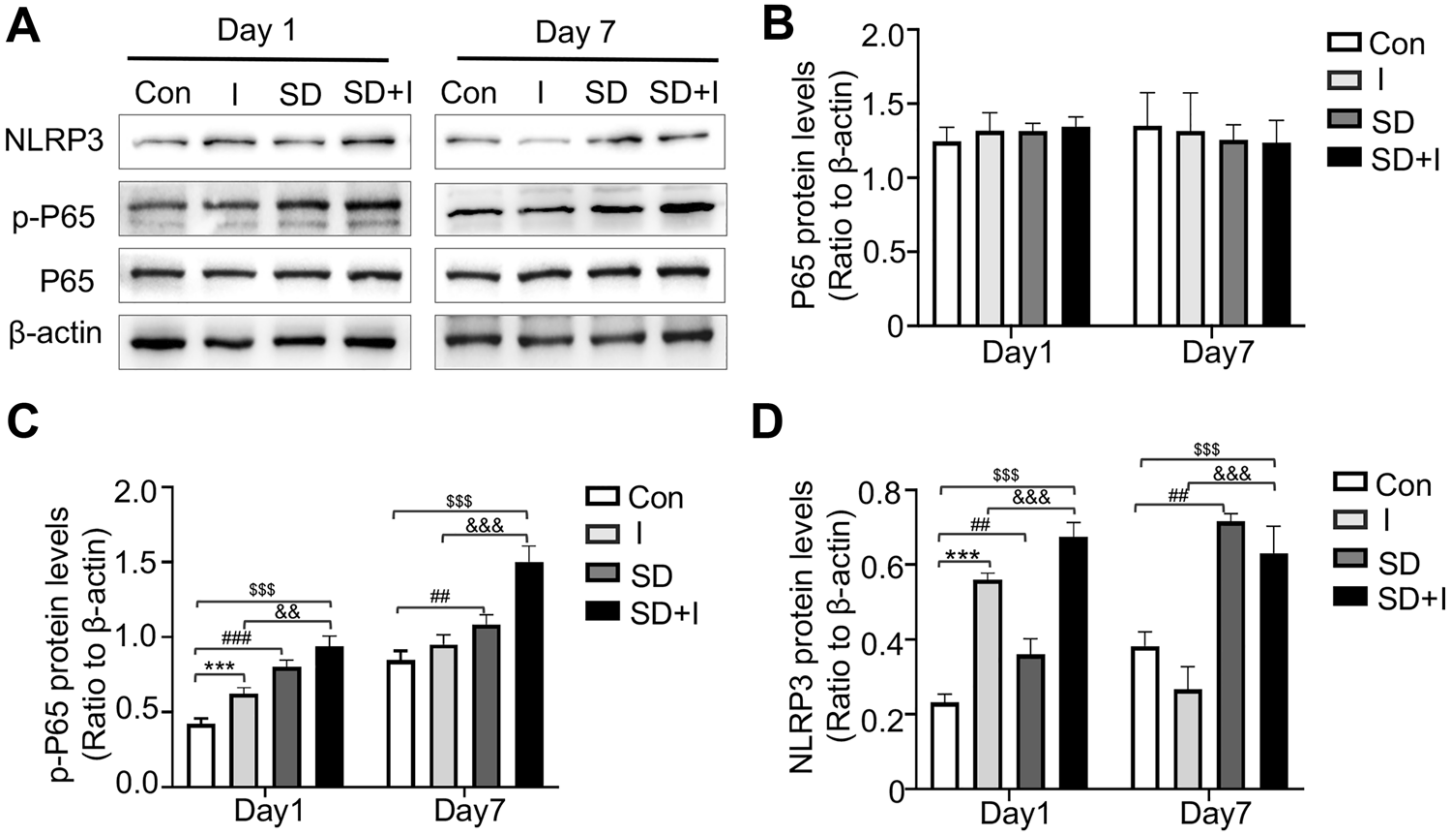
Activation of the NF-κB/NLRP3 signaling after preoperative sleep deprivation in spinal cord. **A** NLRP3, p-P65, and P65 protein expression in spinal cord on day 1 and 7 post-incision measured by western blot. n = 4 per group. **B**–**D** Quantification of P65, p-P65, and NLRP3 protein levels, respectively. Two-way ANOVA for comparisons including more than two groups. Con vs. I: ***p < 0.001; Con vs. SD: ^##^p < 0.01,^###^p < 0.001; Con vs. SD + I: ^$$$^p < 0.001; I vs. SD + I: ^&&^p < 0.01, ^&&&^p < 0.001

Yang et al*.* [19] reported that the NF-κB/NLRP3 signaling pathway mediates pain-induced oxidative stress through the involvement of neuroinflammation. We measured protein levels of P65, p-P65 and NLRP3 in mouse spinal dorsal cord on Day 1 and 7 (Fig. 4A). Interestingly, NLRP3 and p-P65 were significantly upregulated in I group, SD group and SD + I group on Day 1 than those of con group (p-P65: con group vs*.* I group, *p* < 0.001; con group vs*.* SD group, *p* < 0.001; con group vs*.* SD + I group, *p* < 0.001; I group vs*.* SD + I group, *p* < 0.01; F(3,12) = 82.249, *p* < 0.001 for group factor; NLRP3: con group vs*.* I group, *p* < 0.001; con group vs*.* SD group, *p* < 0.01; con group vs*.* SD + I group, *p* < 0.001; I group vs*.* SD + I group, *p* < 0.001; F(3,12) = 151.63, *p* < 0.001 for group factor). Moreover, their protein levels in SD + I group was still significantly higher than those of con group and I group on Day 7, while no significant differences were detected between the latter two groups (p-P65: con group vs*.* SD group, *p* < 0.01; con group vs*.* SD + I group, *p* < 0.001; I group vs*.* SD + I group, *p* < 0.001; F(3,12) = 51.29, *p* < 0.001 for group factor; NLRP3: con group vs*.* SD group, *p* < 0.01; con group vs*.* SD + I group, *p* < 0.001; I group vs*.* SD + I group, *p* < 0.001; F(3,12) = 63.9, *p* < 0.001 for group factor) (Fig. 4B–D). Our findings thus indicated that pretreatment of SD remarkably prolonged the upregulation of p-P65 and NLRP3 after the unilateral hind paw plantar incision, which further caused SD-induced hyperalgesia associated with potentiated activation of microglia and elevated levels of ROS.

### Pretreatment of PBN Alleviates SD-Induced Prolongation of Postsurgical Pain

**Fig. 5:**
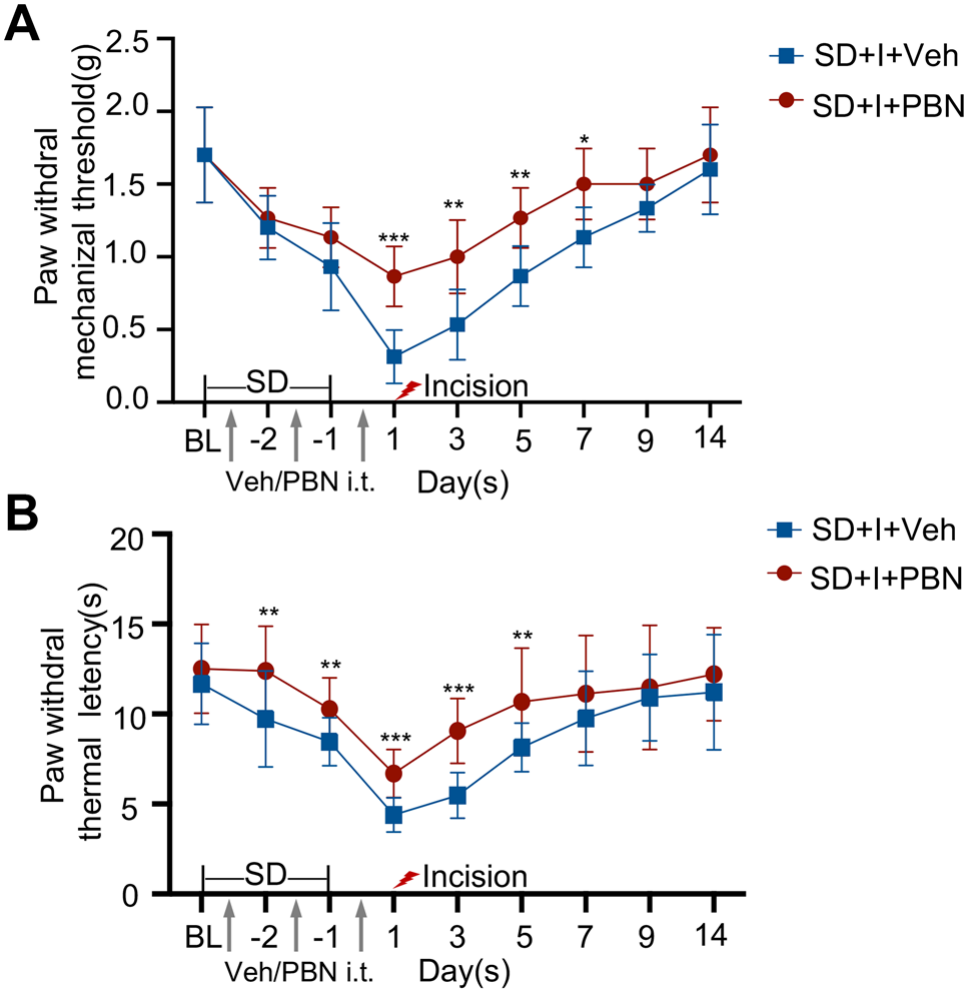
Time-dependent changes in paw withdrawal mechanical threshold and thermal latency in SD-exposed followed hind paw incision mice with pretreatment of intrathecal injection PBN (SD + I + PBN) or saline (SD + I + Veh). **A** Paw withdrawal thresholds in response to mechanical stimulation during SD (Day -2 and -1) and on the days post-incision (Day 1, 3, 5, 7, 9, and 14). **B** Paw withdrawal to heat stimulation in corresponding time points. One-way ANOVA followed by Bonferroni test was performed. n = 8 per group. SD + I + Veh vs. SD + I + PBN: *p < 0.05, **p < 0.01, ***p < 0.001

To confirm the involvement of ROS in SD-induced prolongation of postsurgical pain, the ROS inhibitor PBN (100 µg/5 µl) was injected into mouse intrathecal space during SD period and the day before making the incision. Pretreatment of PBN significantly enhanced PWMT on Day 1, 3, 5, and 7 (SD + I + Veh group vs*.* SD + I + PBN group, *p* < 0.001, *p* < 0.01, *p* < 0.01, and *p* < 0.05, respectively) (Fig. 5A). Compared with SD + I + Veh group, PWLT in SD + I + PBN group was slightly elevated during SD period, which strongly increased on postsurgical Day 1, 3, and 5 (SD + I + Veh group vs*.* SD + I + PBN group, *p* < 0.01, *p* < 0.01, *p* < 0.001, *p* < 0.001, and *p* < 0.01, respectively) (Fig. 5B).

### Pretreatment of PBN Reduces ROS Levels in Mouse Spinal Dorsal Cord, and Suppresses Microglia Activation and NLRP3 Inflammasome Activity

**Fig. 6:**
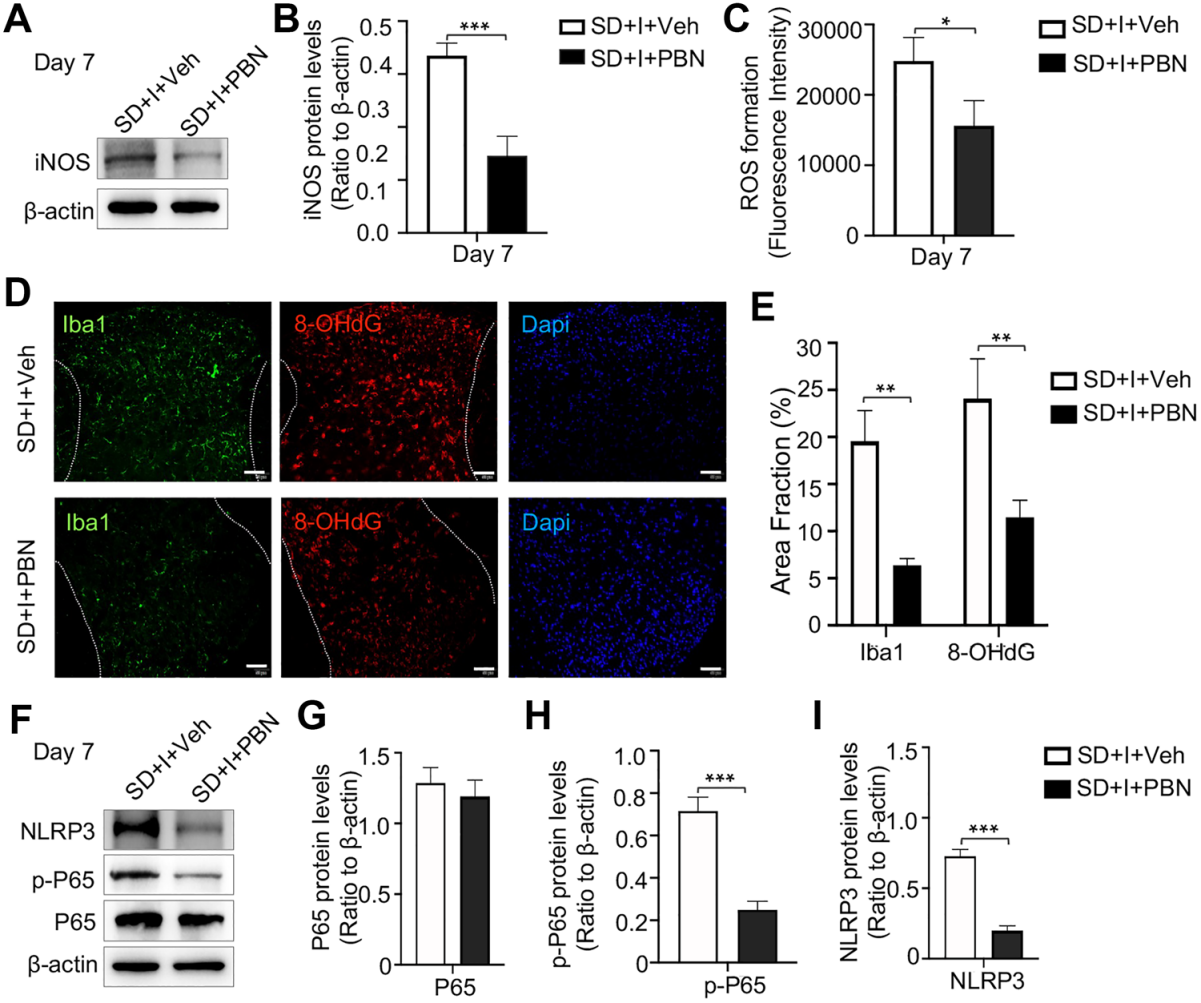
Effect of PBN intrathecal injection on oxidative stress, microglia activation, DNA damage, and NF-κB/NLRP3 activity in spinal dorsal cord of SD-exposed followed hind paw incision mice. **A** iNOS expression in spinal cord on day 7 post-incision measured by western blot. n = 4 per group. **B** Quantification of iNOS protein level. **C** Fluorescence intensity of ROS levels in spinal cord tissue. n = 3 per group. **D** Images of immunostaining for Iba1and 8-OHdG in lumbar spinal dorsal horn ipsilateral to incision were captured in group SD + I + Veh and group SD + I + PBN on Day 7 post-incision. Scale bar = 50 μm. n = 4 per group. **E** Quantification of Iba1 and 8-OHdG fluorescence intensity, respectively. **F** NLRP3, p-P65, and P65 protein expression in spinal cord on day 1 and 7 post-incision measured by western blot. n = 4 per group. **G**–**I** Quantification of P65, p-P65, and NLRP3 protein levels, respectively. Two-way ANOVA for comparisons including more than two groups; One-way ANOVA for two group comparisons; SD + I + Veh vs. SD + I + PBN: **p < 0.01

To further investigate the role of PBN in alleviating SD-induced prolongation of postsurgical pain, we measured protein level of iNOS and ROS levels in mouse spinal dorsal cord. Compared with those of controls, intrathecal injection of PBN significantly downregulated iNOS and ROS levels on Day 7 (SD + I + Veh group vs*.* SD + I + PBN group, *p* < 0.001 and *p* < 0.01, respectively) (Fig. 6A–C). Moreover, microglial activation and DNA damage were significantly alleviated in mice of SD + I + PBN group than those of SD + I + Veh group on Day 7 (SD + I + Veh group vs*.* SD + I + PBN group, *p* < 0.05 and *p* < 0.05, respectively) (Fig. 6D, E). As expected, pretreatment of PBN effectively downregulated NLRP3 and p-P65 in mouse spinal dorsal cord (SD + I + Veh group vs*.* SD + I + PBN group, *p* < 0.001 and *p* < 0.001, respectively) (Fig. 6F–I). Overall, pretreatment of PBN remarkably alleviated SD-induced hyperalgesia by suppressing microglia activation and NLRP3 inflammasome activity in the spinal dorsal cord.

## Discussion

In the present study, we carried out a 48-h continuous SD before the hind paw incision surgery model to mimic preoperative sleep disorders in postoperative patients. Our findings demonstrated that SD prolonged postsurgical pain via inducing microglia activation, increasing ROS levels and upregulating p-P65 and NLRP3 in the spinal dorsal cord. Pretreatment of a non-specific ROS scavenger, PBN, by intrathecal administration, effectively alleviated SD-associated pathological development through enhancing PWMT and PWTL, inhibiting microglia activation and suppressing ROS levels via the NF-κB/NLRP3 signaling pathway.

CPSP is a negative outcome of over 3-month pain after surgical procedures [20]. The incidence of CPSP remains high (3–56%), which remains often undermanaged [5]. Most surgical patients have normal pain perception before surgery, but sleep disorders in perioperative period, especially those with critical surgery [21, 22]. Emerging evidence has highlighted the critical role of the perioperative sleep disturbances and sleep disorders in the development of CPSP [23]. Luo et al. [24] revealed that the preoperative Pittsburgh sleep quality index (PSQI) scores are significantly correlated with pain scores at 3 months after total knee and hip arthroplasty, as well as the consumption of analgesics after joint replacement. The lower sleep efficiency the night before surgery of breast cancer patients is associated with a higher postoperative self-reported pain [25]. Poor sleep quality is also linked with the incidence of severe postoperative pain and increased intake of analgesics after delivery [26]. Our data showed that PWMT and PWLT significantly decreased in mice intervened by 48-h continuous SD, which persisted at least for 3 days. A previous study reported that rapid eye movement sleep disturbance (REMSD) 6 h daily for consecutive 3 days does not influence the basal pain sensitivity, including responses to heat, cold and mechanical stimuli [27]. However, REMSD for consecutive 2–4 days in rats markedly reduces PWLT to the heat stimuli [28]. Our findings showed that SD significantly prolonged the recovery of incision-induced decreases in PWMT and PWLT. The underlying mechanism, however, remained unclear.

Previous studies indicate that SD induces oxidative stress in many tissues as well as subsequent decrease in antioxidant capacity [8], and increased ROS in the spinal cord followed by neuroinflammation [29]. Here, we found increased activation of microglia in spinal cord of SD mice, which indicated that inflammatory reaction occurred after SD exposure. Also, SD is capable of affecting DNA integrity by prolonging oxidative stress [30–32]. It causes single-strand breaks by attacking deoxyribose through excessively produced ROS. The following response of cells to repair single-strand breaks further results in DNA double-strand breaks and delays DNA repair [32]. SD-induced oxidative stress directly stimulates DNA damage during the pain [33]. Consistently, our results showed increased levels of ROS and 8-hydroxyguanosine (8-OHdG; a modified base formed when DNA is attacked by hydroxyl radicals) in mouse spinal dorsal cord of SD group and SD + I group on Day 1 and 7. Previous studies revealed that excessive ROS generation is associated with endoplasmic reticulum (ER) stress, which induces inflammatory response by activating the nuclear factor-kappa B (NF-κB) signaling pathway [34]. Oxidative stress is involved in the activation of NLRP3 inflammatory corpuscles, which is an important component of innate immunity involved in the process of inflammatory diseases of the CNS [35–37]. Latest studies have shown that NLRP3 is up-regulated in both neuropathic and inflammatory pain models, the activation of which, is also involved in the occurrence and maintenance of postoperative pain [38]. Activation of the NLRP3 inflammasome has been linked to NF-kB activation and inflammatory cytokine secretion [39]. The NF-κB/NLRP3 signaling pathway is responsible for mediating hypobaric hypoxia-induced brain injury and traumatic brain injury-induced neuroinflammation [40, 41]. In our study, preoperative SD remarkably activated NLRP3 after making the incision, suggesting the potential role of the NF-κB/NLRP3 signaling pathway in CPSP.

Microglia are of significance in the generation and maintenance of hyperalgesia [42]. Spinal microglia participate in the occurrence and maintenance of chronic pain by regulating the immune response in the spinal cord [43]. Significantly activated substantia nigra microglia are detected in patients with sleep disorders [44]. Our study consistently detected microglia activation in the spinal dorsal cord of SD-induced mice, suggesting that microglial activation was involved in the prolongation of postsurgical pain.

To sum up, the increase in ROS after the short-term SD resulted in DNA damage, followed by microglia sensitization at the spinal cord level. Surgical stress further aggravated DNA damage and DNA escape to the cytoplasm, where NLRP3 was activated and induced CPSP by mediating the inflammatory response. Intrathecal injection of PBN during SD effectively alleviated the incisional pain after SD by reducing ROS levels and DNA damage. Therefore, eliminating ROS is expected to be a promising therapeutic strategy to SD-induced CPSP.

## Data Availability

The data that support the findings of the current study are available from the corresponding author Zhengliang Ma on reasonable request.
